# Quality of Mobile Phone and Tablet Mobile Apps for Speech Sound Disorders: Protocol for an Evidence-Based Appraisal

**DOI:** 10.2196/resprot.6505

**Published:** 2016-11-29

**Authors:** Lisa M Furlong, Meg E Morris, Shane Erickson, Tanya A Serry

**Affiliations:** ^1^ School of Allied Health Discipline of Speech Pathology La Trobe University Bundoora Australia; ^2^ Healthscope Northpark Private Hospital & La Trobe University Bundoora Australia; ^3^ Centre for Sport & Exercise Medicine Research Bundoora Australia

**Keywords:** speech therapy, medical informatics, rehabilitation, mHealth, speech sound disorders

## Abstract

**Background:**

Although mobile apps are readily available for speech sound disorders (SSD), their validity has not been systematically evaluated. This evidence-based appraisal will critically review and synthesize current evidence on available therapy apps for use by children with SSD.

**Objective:**

The main aims are to (1) identify the types of apps currently available for Android and iOS mobile phones and tablets, and (2) to critique their design features and content using a structured quality appraisal tool.

**Methods:**

This protocol paper presents and justifies the methods used for a systematic review of mobile apps that provide intervention for use by children with SSD. The primary outcomes of interest are (1) engagement, (2) functionality, (3) aesthetics, (4) information quality, (5) subjective quality, and (6) perceived impact. Quality will be assessed by 2 certified practicing speech-language pathologists using a structured quality appraisal tool. Two app stores will be searched from the 2 largest operating platforms, Android and iOS. Systematic methods of knowledge synthesis shall include searching the app stores using a defined procedure, data extraction, and quality analysis.

**Results:**

This search strategy shall enable us to determine how many SSD apps are available for Android and for iOS compatible mobile phones and tablets. It shall also identify the regions of the world responsible for the apps’ development, the content and the quality of offerings. Recommendations will be made for speech-language pathologists seeking to use mobile apps in their clinical practice.

**Conclusions:**

This protocol provides a structured process for locating apps and appraising the quality, as the basis for evaluating their use in speech pathology for children in English-speaking nations.

## Introduction

### mHealth

At the beginning of 2016, there were an estimated 7.4 billion mobile subscriptions worldwide, of which 3.4. billion were mobile phone subscriptions [1]. With the exponential increase in the use of mobile devices globally, there is increased interest from speech-language pathologists (SLPs), clients, and their families regarding how mobile apps can be used to enhance the management of childhood speech sound disorders (SSD).

The Global Observatory for eHealth of the World Health Organization defines mHealth as “medical and public health practice supported by mobile devices, such as mobile phones, patient monitoring devices, personal digital assistants and other wireless devices” [2]. mHealth apps are software programs developed for handheld devices used to provide advice around prevention of disease and healthy living, to screen and support self-management of chronic diseases (eg, asthma, diabetes, cardiovascular disease), to support adherence to treatment regimes, promote behavior change, educate patients, and to offer providers and consumers access to health care–related information and services [3-7].

### Mobile Apps for Speech Sound Disorders

For SLPs, mobile apps for SSD are becoming prevalent in clinical practice [8]. There is an opportunity for mobile apps to assist in the clinical management of SSD, for example, by supplementing speech therapy, increasing practice time, offering biofeedback or information relating to the accuracy of the user’s attempt, enhancing families’ engagement with speech therapy, and by strengthening SLPs’ ties with clients by offering them extended treatment throughout the day [8]. Mobile apps are arguably cost effective, accessible, and convenient [7]. For SLPs, apps allow access to otherwise expensive equipment like decibel readers and voice recorders, at an affordable price. Apps are easy to store and transport, and are durable, unlike traditional paper-based resources and games, which are subject to wear and tear. Apps also save time, offering SLPs the convenience of automatic record keeping and monitoring of progress (eg, tallies/scoring) as well as by reducing SLPs’ preparation time. Many apps also have the option to customize or personalize particular features (eg, by adding personal photos and pictures).

However, the magnitude and rapid explosion of available apps poses a challenge for how SLPs and clients can find them and determine which ones to use. Due to the constraints of clinical practice, SLPs do not always have the time to find suitable apps or to critically appraise their quality. The resources available to SLPs and other consumers (eg, parents, caregivers, teachers) to choose or recommend particular apps can be scant. Instead, SLPs and other consumers may have to rely on subjective ‘star rating systems’ or user reviews, which may not accurately reflect the efficiency, effectiveness, or quality of the app. They are unlikely to be able to ascertain the extent to which the app was developed in reference to principles of evidence-based practice [7].

Children with SSD have “any combination of difficulties with perception of speech sounds, articulation/motor production, and/or phonological representation of speech segments (consonants and vowels), phonotactics (syllable and word shapes), and prosody (lexical and grammatical tones, rhythm, stress and intonation)” [9]. To promote gains in speech production accuracy and reduce the risk of later social, academic, or emotional difficulties [10,11], these children benefit from frequent, effective, and intensive therapy [12]. The efficiency and effectiveness of therapy is dependent on a range of factors relating to service delivery (eg, therapy setting, therapy schedule, family support and involvement), client factors (eg, age, motivation, attention, self-awareness), and the treating SLP (eg, experience, expertise) [13].

The use of mobile apps as a technology-based intervention has the potential to influence some of these factors. They have the potential to influence or enhance the efficiency and effectiveness of traditional therapy. In other domains of speech pathology, the use of technology-based interventions has been shown to increase intervention intensity [14,15], which may allow clients to reach expected outcomes earlier but without the need for simultaneous increased direct contact time with a SLP. Technology-based interventions allow for practice between therapy sessions in a variety of settings [16,17], which may be particularly beneficial in generalizing communication skills. Using technology has also been shown to increase enjoyment, motivation, and compliance for therapy both within [18,19] and outside of the clinic [20].

Such benefits have been demonstrated in recent studies, which have specifically evaluated health-related mobile apps, for example, for the self-management of chronic conditions (eg, diabetes, asthma, depression) [21], for treatment adherence in patients with bipolar disorder [22], and for the provision of lifestyle interventions for weight-loss in cancer survivors [23].

An initial step in understanding how mobile apps can be used in to enhance the management of SSD is to identify mobile apps that are currently available for this client population. The evidence base for use of mobile apps in the management of childhood SSD is sparse. Despite there being a number of Web-based app catalogues, reviews, blogs, and recommendations for the use of mobile apps in speech pathology (typically authored by SLPs); to the authors’ knowledge, there are no published reviews on this topic. While the use of mHealth holds promise for this client population, the feasibility of implementing mHealth depends on the quality of available apps.

### Aim

We provide the protocol for a systematic review of currently available mobile apps for children with SSD. A systematic process of selection and evaluation of apps from the app stores of the 2 largest operating platforms will take place. Two certified practicing SLPs will evaluate the included apps using a structured quality appraisal tool. This shall enable a quality assessment of available apps across the indicators of engagement, functionality, aesthetics, information quality, subjective quality, and perceived impact [24]. This content will be summarized and presented in a way that aids decision making for both SLPs and consumers when selecting an app for this client population.

## Methods

### Design

The systematic review will be conducted in accordance with the Preferred Reporting Items for Systematic reviews and Meta-Analyses (PRISMA). PRISMA is an evidence-based minimum set of items designed to help authors improve the reporting of systematic reviews and meta-analyses. Since its inception, it has been applied to other types of research (eg, evaluations of interventions) and has recently been applied in a review of health-related mobile apps [25].

### Sources, Search Terms, and Search Strategy

The Google Play store and Apple iTunes Store will be searched. These 2 stores have been selected because they are linked to the 2 most widely used operating platforms, Android and iOS. The selection of these operating platforms is based on their substantial share in the mobile app market. According to the International Data Corporation shipment figures, the most popular operating platforms by market share in the second quarter of 2015 were Android (82.8%) and iOS (13.9%) [26]. While this information is specific to the worldwide mobile phone market, these data support our rationale to use these 2 operating platforms in our review of mobile apps for both mobile phones and tablets. Previous reviews evaluating health-related mobile apps have also sourced apps solely from these 2 operating platforms [7,27,28].

Using the Web interface of the Google Play and Apple iTunes stores, a list of defined terms will be entered into the search fields. This method was chosen following phone consultations with specialists at Apple Support and Google Play. This search method has also been used in other studies evaluating mobile apps in the areas of asthma [27], bipolar disorder [1,7], and health care–associated infection prevention [28]. The search terms were defined in consultation with experts in the field of childhood SSD and app specialists from Apple Support and Google Play. In the Apple iTunes Store, the search terms will be entered within 2 separate categories: apps for iPhone and apps for iPad. In the Google Play store, search terms will be entered only once as there is no capacity to search by device. Rather, apps will be later categorized according to device compatibility.

**Figure 1:**
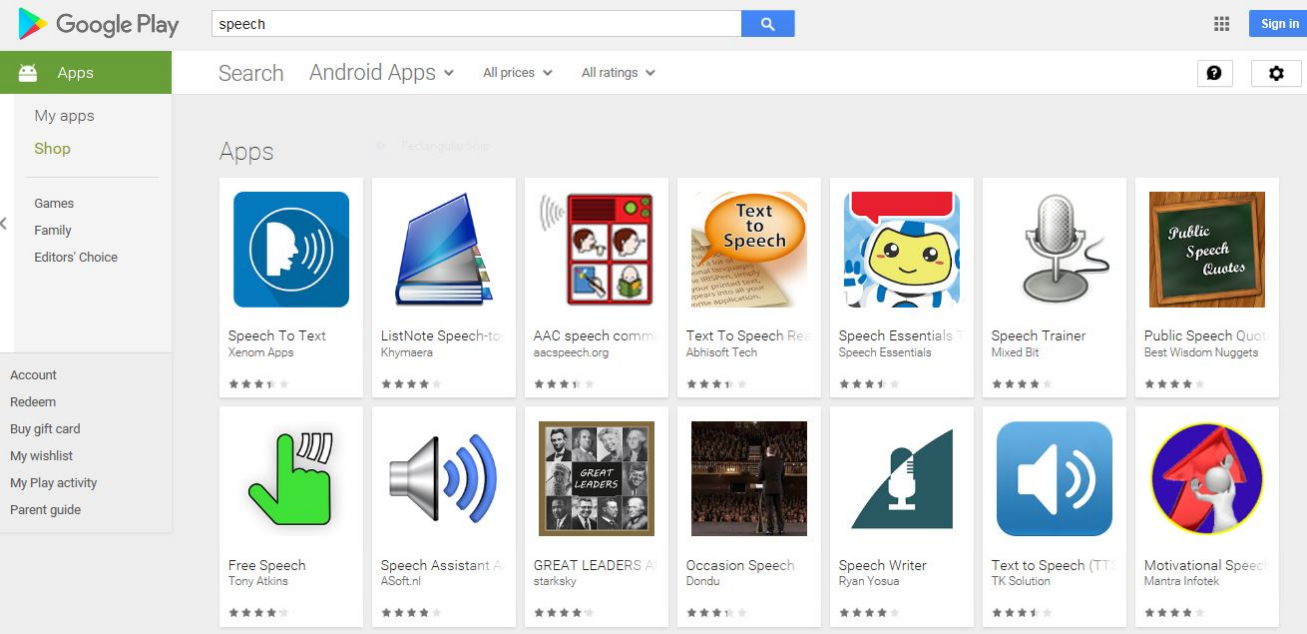
Example search of Google Play store. Google and the Google logo are registered trademarks of Google Inc., used with permission.

Search terms include relevant synonyms and layperson terms to account for the wide variety of consumers accessing the app stores. The search terms are: “speech, phonology, phonological, articulation/artic, talk, pronunciation, speak, say, chat, speech therapy, speech pathology.” Figure 1 shows an example search of the Google Play store.

### Eligibility Criteria and App Selection

The aim of the selection process is to find mobile apps that can be used in the management of childhood SSD. This process will be performed using the following eligibility criteria: provision of activities or tasks requiring production of speech by the user (ie, not just listening or auditory discrimination tasks), developed for speakers of English, free or paid, running on Android or iOS, and available on mobile phone or tablet. Exclusion criteria are: apps that provide speech production training for second language learners (ie, accent modification), apps that teach foreign languages, speech to text/text to speech apps, alternative and augmentative communication apps, apps designed for clients with voice disorders, apps designed to develop receptive or expressive language skills (eg, following directions, semantics, syntax), and apps providing assessment only. To screen apps for inclusion in the review, a 3-step process will be used: (1) collation, (2) broad screening, and (3) focused screening.

### Collation of Titles Generated by the Search

A research assistant will enter the defined search terms into the search field of the Web interface for both the Google Play and Apple iTunes stores. The titles and icons of all resulting apps of all resulting apps will be entered into a spreadsheet, organized according to the app store in which they were located. Duplicate applications using different search terms from the same app store will be removed. For apps that are compatible with both tablets and mobile phones, both will be included to investigate the differences that may exist between the 2 versions.

### Broad Screening

First, manual inspection of titles within the spreadsheet will be screened independently for inclusion criteria by 2 independent reviewers; both certified practicing SLPs. Following this, reviewers will meet and each present a list of apps for inclusion and subsequent screening. Discrepancies between the lists will be identified and discussed until consensus can be reached. If consensus cannot be reached, consultation with a third reviewer will occur. Majority rule will be used to determine inclusion.

### Focused Screening

**Figure 2:**
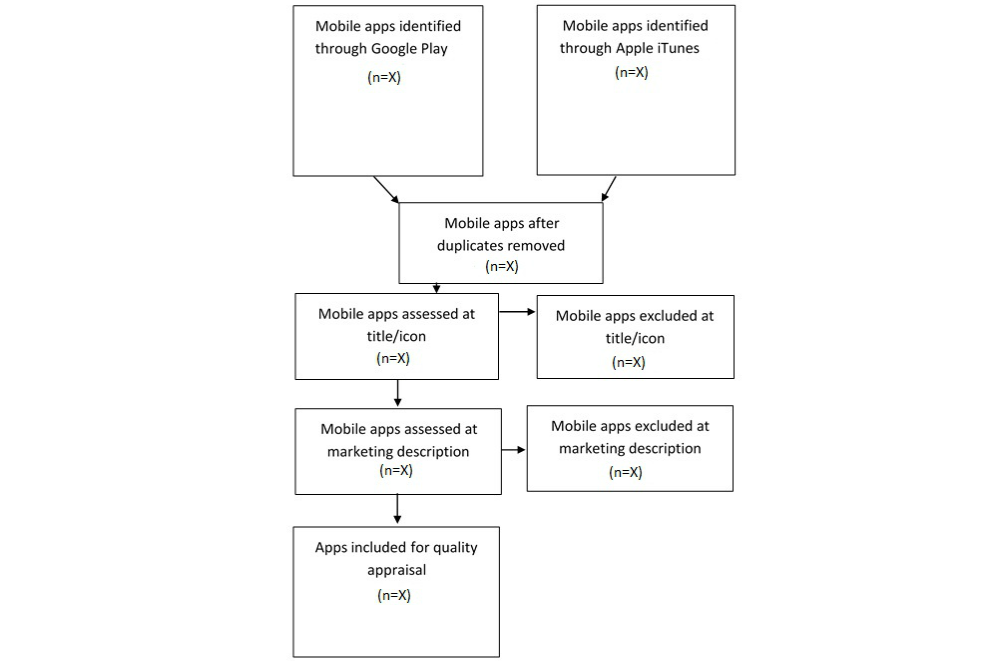
Search and selection process.

The research assistant involved in step 1 of the screening process will return to the Google Play and Apple iTunes stores to extract the marketing description of all apps included at broad screening so that further screening can take place. Marketing descriptions will be entered into the same spreadsheet used in step 2, alongside the app title, icon, and source (app store). The same 2 reviewers involved in step 2 (broad screening) will independently review the full marketing description of the included apps within the spreadsheet. Apps will be selected based on the eligibility criteria described above. A list of apps for qualitative assessment will be compiled independently by each reviewer. Reviewers will meet to discuss apps for final inclusion. Consensus will be reached through discussion. If consensus cannot be reached, a third reviewer will be consulted. Majority rule will be used to determine inclusion. Apps meeting inclusion criteria at this final screening stage will be downloaded for further evaluation. Figure 2 displays the search and selection strategy

### Data Extraction

Apps meeting inclusion criteria will be downloaded onto 4 devices: a Samsung Galaxy Tab A 8.0 WiFi 16GB (Android Version 5.0 [Lollipop]), an iPad 3 (iOS Version 9.3.4), an Android phone (to be specified), and iPhone 5S (iOS Version 9.3.4) for complete assessment. App classification data will be extracted from the marketing description in the app stores by the first author (involved in the screening process) and entered into a spreadsheet: app name and version, time of latest update, app update frequency (average), number of updates, rating current version/all versions, developer, number of ratings for current versions/all versions, cost (basic version/upgrade version), platform, marketing description [24], and device compatibility. A second reviewer will independently extract the same data for 10% of the included apps. Interrater reliability for data extraction will be determined by comparing the 15 data points.

### Data Analysis

The quality of the apps will be evaluated by the 2 reviewers involved in the screening process, using the Mobile Application Rating Scale (MARS) [24]. Both reviewers are certified practicing SLPs with clinical experience in the management of childhood SSD. The MARS is a tool for assessing the quality of mHealth apps. It was developed by an expert multidisciplinary team from the Institute of Health and Biomedical Innovation and Queensland University of Technology, as part of an Australian Government Initiative [24]. The MARS evaluates app quality using a 5-point scale (1-inadequate, 2-poor, 3-acceptable, 4-good, 5-excellent) across the indicators of: engagement, functionality, aesthetics, information quality, subjective quality, and perceived impact [24]. The MARS provides a total mean score for the overall quality of an app. The highest potential mean score is 145 (29 questions across 6 indicators). Mean scores can also be calculated for each indicator to clearly identify the strengths and weaknesses of the app.

In its 2014 pilot study, the MARS total score assigned to the 50 apps included in the pilot, achieved high levels of interrater reliability (two-way mixed intraclass correlation coefficient [ICC]=.79; 95%CI 0.75-0.83) [24]. Concurrent validity was determined by comparison of the MARS total score with the Apple iTunes App Store star rating for 15 of 50 apps involved in the pilot. A moderate correlation between the MARS total score and the Apple iTunes star rating (r_15_=.55, *P*<.05) [24] was reported.

In accordance with the recommendations provided by the MARS developers, the 2 raters involved in the quality assessment of the included mobile apps will complete the Web-based MARS training module prior to the quality assessment taking place. Completion of this training module will ensure that the raters understand the purpose of the MARS and how to use it. Completion of this training module should also improve interrater reliability. In keeping with the guidelines provided by the MARS developers, assignment of app quality ratings will initially be piloted with 5 apps not included in the review to establish interrater reliability [24]. A two-way mixed ICC will be used to determine how consistent the 2 raters are, relative to each other. An ICC above 0.75 is indicative of “good reliability” [29] and this will need to be achieved before the apps included in the review are appraised. If this level of reliability is not achieved following piloting of the MARS with these 5 apps, further training around the use of the MARS will take place. This may involve a repeat viewing of the Web-based training module, discussion around the MARS indicators, consultation with the MARS developers, and/or further piloting of the MARS with additional apps. Following appraisal of the included apps, the two-way mixed ICC will again be calculated for the MARS total score and each MARS subscale.

The Pearson product-moment coefficient of correlation will be calculated to determine whether a correlation exists between the MARS score assigned by the certified practicing SLPs to each app and the star ratings assigned by users of each app in the Apple iTunes and Google Play stores.

### Data Synthesis

The results will relate to the data extracted and quality assessment performed on the included apps. Descriptive and technical information relating to the included apps will be presented in a table and summarized narratively within the text. Graphs and tables will enable comparison of the quality of included apps across the MARS indicators of engagement, functionality, aesthetics, information quality, subjective quality and perceived impact. The 10 mobile apps achieving the highest total mean score for quality will be described in detail to provide consumers with a list of the Top 10 Mobile Apps for Children with SSD.

## Results

Searching of the 2 app stores is currently underway. Broad and focused screening will commence toward the end of 2016. Data extraction and quality appraisal of the selected apps will commence in 2017.

## Discussion

### Implications

This protocol paper presents and justifies the methods for a systematic review of mobile apps for children with SSD. The aim of the systematic review is to identify the types of apps currently available for Android and iOS mobile phones and tablets and to critique their design features and content using a structured quality appraisal tool.

### Conclusions

Amid a plethora of mobile apps for children with SSD, it is becoming increasingly difficult for SLPs to identify high quality apps for clinical use. This protocol describes a systematic search, selection, and appraisal process of mobile apps for children with SSD. This review will provide descriptive and technical information in addition to a quality assessment for the included apps. These results will assist SLPs in making an informed choice when selecting and recommending apps for the clinical management of this client population.

## Abbreviations

ICC: intraclass correlation coefficient
PRISMA: preferred reporting items for systematic reviews and meta-analyses
MARS: mobile application rating scale
SLP: speech-language pathologist
SSD: speech sound disorders
